# Tracing of the Bile-Chemotactic Migration of Juvenile *Clonorchis sinensis* in Rabbits by PET-CT

**DOI:** 10.1371/journal.pntd.0001414

**Published:** 2011-12-13

**Authors:** Tae Im Kim, Won Gi Yoo, Byung Kook Kwak, Ju–Won Seok, Sung–Jong Hong

**Affiliations:** 1 Department of Medical Environmental Biology, Chung-Ang University College of Medicine, Seoul, Republic of Korea; 2 Division of Malaria and Parasitic Diseases, National Institute of Health, Korea Centers for Disease Control and Prevention, Osong, Chungbuk, Republic of Korea; 3 Department of Radiology, Chung-Ang University College of Medicine, Dongjak-gu, Seoul, Republic of Korea; 4 Department of Nuclear Medicine, Chung-Ang University College of Medicine, Dongjak-gu, Seoul, Republic of Korea; Khon Kaen University, Thailand

## Abstract

**Background:**

Adult *Clonorchis sinensis* live in the bile duct and cause clonorchiasis. It is known that the *C. sinensis* metacercariae excyst in the duodenum and migrate up to the bile duct through the common bile duct. However, no direct evidence is available on the *in vivo* migration of newly excysted *C. sinensis* juveniles (*Cs*NEJs). Advanced imaging technologies now allow the *in vivo* migration and localization to be visualized. In the present study, we sought to determine how sensitively *Cs*NEJs respond to bile and how fast they migrate to the intrahepatic bile duct using PET-CT.

**Methodology/Principal Findings:**

*Cs*NEJs were radiolabeled with ^18^F-fluorodeoxyglucose (^18^F-FDG). Rabbits with a gallbladder contraction response to cholecystokinin-8 (CCK-8) injection were pre-screened using cholescintigraphy. In these rabbits, gallbladders contracted by 50% in volume at an average of 11.5 min post-injection. The four rabbits examined were kept anesthetized and a catheter inserted into the mid duodenum. Gallbladder contraction was stimulated by injecting CCK-8 (20 ng/kg every minute) over the experiment. Anatomical images were acquired by CT initially and dynamic PET was then carried out for 90 min with a 3-min acquisition per frame. Twelve minutes after CCK-8 injection, about 3,000 ^18^F-FDG-labeled *Cs*NEJs were inoculated into the mid duodenum through the catheter. Photon signals were detected in the liver 7–9 min after *Cs*NEJs inoculation, and these then increased in the whole liver with stronger intensity in the central area, presenting that the *Cs*NEJs were arriving at the intrahepatic bile ducts.

**Conclusion:**

In the duodenum, *Cs*NEJs immediately sense bile and migrate quickly with bile-chemotaxis to reach the intrahepatic bile ducts by way of the ampulla of Vater.

## Author Summary

*Clonorchis sinensis* adults habituating in the bile duct cause clonorchiasis endemic in East Asian countries, in which about 15–20 million people are supposedly infected. It has previously been reported that *C. sinensis* metacercariae excyst in the duodenum and that the juvenile flukes migrate to the bile duct through the ampulla of Vater in 4–7 hours. Recently advanced imaging technologies have enabled visualization of movements and localizations of parasites in mammalian hosts. From present study, we found the following: newly excysted *C. sinensis* juveniles (*Cs*NEJs) were efficiently *in vitro* radiolabeled with ^18^F-FDG since *Cs*NEJs have glucose transporters; CCK-8-induced gallbladder contraction was various rabbit to rabbit; *Cs*NEJs promptly recognized bile and migrated up the duodenum to reach the intrahepatic bile ducts by way of the ampulla of Vater and the common bile duct as early as 7–9 minutes after inoculation. Some *Cs*NEJs responding slowly to the bile delayed arriving at the distal bile capillaries. It was visualized for the first time that the *Cs*NEJs migrate quickly within 10–20 minutes from the duodenum to the intrahepatic bile duct. These findings provide fundamental information on the migration of parasites living in the biliary passages of mammals.

## Introduction

Human *Clonorchis sinensis* infections are endemic in East Asia countries, such as China, Vietnam, and Korea, where 15–20 million people are estimated to be infected [1]. In South Korea, clonorchiasis is currently the most prevalent parasitic infection and estimated to infect 1.3 million people [2]. *C. sinensis* infected patients suffer from abdominal pain, hepatomegaly, obstructive jaundice, indigestion, and complications of cholecystitis, cholelithiasis, and cholangiocarcinoma [3], [4]. Furthermore, recently, *C. sinensis* was categorized as a Group 1 biological carcinogen by the International Agency for Research on Cancer [5].

Humans are the final host and become infected by eating freshwater fish containing *C. sinensis* metacercariae. Ingested metacercariae excyst in the duodenum due to trypsin stimulation [6], and the newly excysted *C. sinensis* juveniles (*Cs*NEJs) migrate to the intrahepatic bile duct. The migration route of *Cs*NEJs has been previously examined in experimental animals. In rabbit experiments, the common bile duct was first ligated surgically then *C. sinensis* metacercariae were administered to the rabbits through a gastric tube. One month later, adult *C. sinensis* were searched for in the bile ducts, but were not found. Based on this finding it was suggested that *Cs*NEJs migrate through the common bile duct to the intrahepatic bile ducts [7], and this has been taken to be the migration route of *C. sinensis* in mammalian hosts [3].

Parasites such as *C. sinensis* have specific *in vivo* migration routes in their hosts, which could be targeted for development of therapeutic and preventive interventions against parasitic diseases. Furthermore, *in vivo* imaging technologies have been recently developed for the clinical diagnoses of a wide range of diseases, and these techniques have a potential to monitor the movements of *Cs*NEJs.

Molecular imaging has emerged as a discipline at the intersection of molecular biology and *in vivo* imaging. It enables cellular functions to be visualized and molecular processes to be followed in living organisms in a non-invasive manner. Recently, studies on the visualization of live parasite in hosts have been conducted. Using transgenic *Plasmodium* parasites, pre-erythrocytic development was visualized; *Plasmodium* sporozoites entered hepatic cells, developed in a large schizont, and released merozoites in liver [8], [9]. However, these techniques are not applicable to trematodes, because stable transgenic flukes are difficult to be generated.

In mammalian hosts, adult forms of trematodes consume large amounts of glucose to generate and supply energy by running the glycolytic pathway [10]. Adult schistosomes import exogenous glucose, equivalent to their dry body weight every 4 hours from host blood by using glucose transporters in their tegumental membranes [11], [12]. In *C. sinensis*, glucose transporter and Na^+^/glucose co-transporter are expressed abundantly in the adult stage but less so in the metacercarial stage as presented in the *C. sinensis* transcriptome [13]. Adult *C. sinensis* worms uptake glucose to produce energy in the anaerobic environment of the bile duct [14]. Therefore, we expected that *C. sinensis* could be labeled with 2-deoxy-2-[^18^F]fluoro-D-glucose (^18^F-FDG), a glucose analogue used for the radiolabeling and diagnostic imaging of cancer cells [15]. Thus, by *ex vivo* labeling *Cs*NEJs with ^18^F-FDG, we hoped their migration in the final host could be traced *in vivo* by positron emission tomography-computed tomography (PET-CT).

*In vivo* imaging techniques have strong merits for the noninvasive tracing on pathogens moving within tissues of living animals, as they involve minimal manipulation and/or euthanasia of animals, and allow repetitive tracking in same animals. Furthermore, as was found in the present study, these techniques make it possible to monitor the distribution and migration of *Cs*NEJs *in vivo* from the duodenum to the liver or distal bowel. This study was carried out to determine how *Cs*NEJs find their way and how rapidly they migrate to the intrahepatic bile duct by using *in vitro* ^18^F-FDG radiolabeling and PET-CT in a rabbit model.

## Materials and Methods

### 1. Collection of *C. sinensis* metacercariae

Topmouth gudgeons (*Pseudorasbora parva*), the second intermediate host of *C. sinensis*, were purchased at a fish market in Shenyang, Liaoning Province, People's Republic of China. Fishes were ground then digested in artificial gastric juice (8 g of pepsin 1∶10,000 (MP Biochemicals Co., Solon, OH, USA) and 8 ml of concentrated HCl in 1 liter of water) for 2 hr at 37°C [10]. To remove particulate matters, the digested soup was filtered through a sieve of 212 µm mesh. *C. sinensis* metacercariae (135–145 µm×90–100 µm) were then filtered out using seives of 106 and 53 µm meshes and washed thoroughly several times with 0.85% saline. *C. sinensis* metacercariae were collected under a dissecting microscope and stored in phosphate-buffered saline at 4°C until required [10].

### 2. Labeling *Cs*NEJs with radio-isotope

The metacercarial cyst wall of *C. sinensis* is thick and can hinder glucose diffusion. Thus to maximize radiolabeling efficiency, metacercariae were excysted and juvenile worms were liberated from cysts. The *C. sinensis* metacercariae were excysted by treating them with 0.05% trypsin at 37°C for 5 minutes (Gibco, Grand Island, NY, USA) in 1× Locke's solution (150 mM NaCl, 5 mM KCl, 1.8 mM CaCl_2_, 1.9 mM NaHCO_3_), a maintaining medium of *Cs*NEJs [16]. *Cs*NEJs were washed 5 times with 1× Locke's solution, and used immediately. *Cs*NEJs were divided into two groups of 10–270 juveniles each; one was of *Cs*NEJs that excysted just before radiolabeling and the other was of the *Cs*NEJs fasted for 24 hours. The two *Cs*NEJ groups were radio-labeled with ^18^F-FDG by incubating them in 1× Locke's solution containing 74 MBq ^18^F-FDG at 37°C for 15, 30, or 60 min. After washing 3 times with 1× Locke's solution, radioactivity was measured for 10 min using a PET (GEMINI TF, Philips Healthcare, Cleveland, OH, USA). Numbers of *Cs*NEJs were counted and labeling efficiency was calculated as counts per minute (cpm) divided by number of the *Cs*NEJs. Radio-labeling efficiencies of the *Cs*NEJs in both groups were measured 3 times and significant differences were determined using the student's *t*-test.

### 3. Gallbladder contraction in response to cholecystokinin by cholescintigraphy

Rabbits (New Zealand White, male, 2.2–2.5 kg) were purchased from Samtako Bio Korea Inc. (Osan, Korea). Rabbits were cared for and handled according to guidelines issued by Chung-Ang University College of Medicine Animal Facility (an accredited facility) in accordance with AAALAC International Animal Care policy. Animal experiments were approved by the institutional review board of the Chung-Ang University animal facility (CAUMD 09-0024).

Gallbladder contraction and emptying time induced by cholecystokinin-8 (CCK–8) varied from rabbit to rabbit. To select rabbits that responded sensitively to CCK-8, cholescintigraphy and ^99m^Tc-mebrofenin (3-bromo-2,4,6-trimethylphenyl carbamoylmethyl iminodiacetic acid) were used. Briefly, rabbits were fasted for 12 hrs and anesthetized with a 0.47 mg/kg Rompun (xylazine hydrochloride; Bayer Korea, Seoul, Korea) and 12.5 mg/kg Zoletil 50 (Zolazepam and Tiletamine; Virvac Korea, Seoul), intramuscular injection. ^99m^Tc-mebrofenin (74 MBq) in 0.5 ml volume was then administered via an ear vein to each anesthetized rabbit. When full of ^99m^Tc-mebrofenin, gallbladders were stimulated to contract by injecting CCK–8 intravenously at 20 ng/kg every 1 min. A dynamic image was taken every 1 min for 1 hour for each rabbit. All images were obtained with a rotating dual-headed gamma camera equipped with a low-energy, high-resolution collimator (Vertex TM, Philips Healthcare, Cleveland, OH, USA) using a 256×256-pixel matrix at an energy range of 20% at 140 keV.

### 4. *In vivo* imaging of migration of the *Cs*NEJs using PET-CT

**Figure 1:**
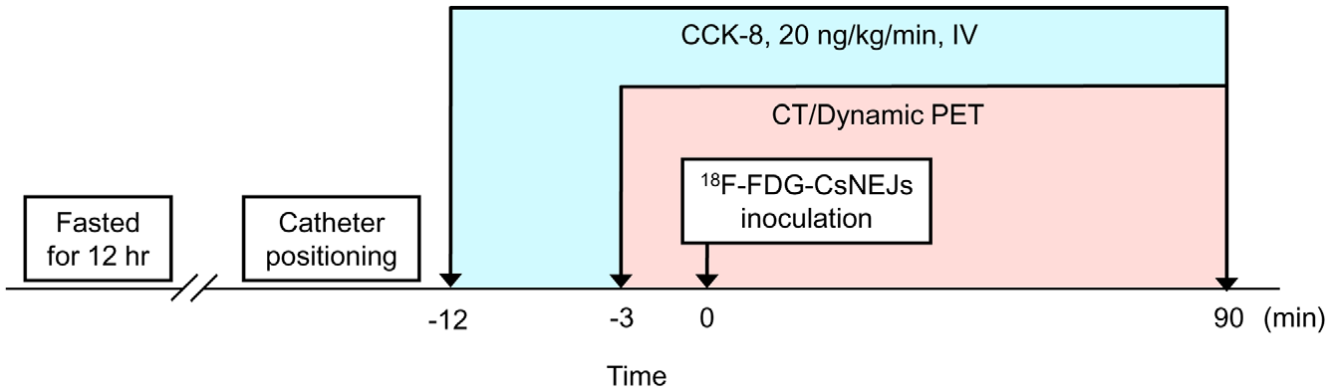
Flow-chart of the PET-CT imaging of the migration of newly excysted *Clonorchis sinensis* juveniles (*Cs*NEJs) in rabbits.

Fresh *Cs*NEJs (n = ∼3,000) were radio-labeled with ^18^F-FDG by incubating them in a maintaining medium containing 74 MBq ^18^F-FDG at 37°C for 15 min. *Cs*NEJs were washed 3 times with 1× Locke's solution and then placed in 500 µl of 1× Locke's solution. The procedure was conducted as follows (Figure 1). A rabbit sensitive to CCK-8 was anesthetized with 0.47 mg/kg Rompun and 12.5 mg/kg Zoletil 50 by intramuscular injection and placed in restraints in a supine position on a plastic board. A catheter (5F Simmons II, Cook Co., Bloomington, IN, USA), equipped with a guidewire (0.035″ Radifocus®, Terumo, Tokyo), was inserted through the animal's mouth and its end positioned in the mid duodenum under guidance (Axiom Artis; Siemens, Erlangen, Germany). The rabbit was then moved with the catheter *in situ* and placed in PET-CT bed. To stimulate gallbladder contraction and bile juice release, 20 ng/kg of CCK–8 was injected intravenously every minute over this experiment [17]. After 12 minutes of CCK-8 injection, ^18^F-FDG-labeled *Cs*NEJs in 500 µl of 1× Locke's solution were introduced into the mid duodenum through the catheter; residual *Cs*NEJs in the catheter were flushed into the duodenum with 0.5 ml of 1× Locke's solution. One transmission CT image was obtained before the introduction of the ^18^F-FDG-labeled *Cs*NEJs and a dynamic PET scan then was performed over 90 min with a 3-min acquisition per frame. Finally, one static PET image was scanned for 10 min. This procedure is depicted schematically as a flow-chart in Figure 1.

All photon data were collected using a dedicated PET-CT scanner. PET images were reconstructed after applying CT-based attenuation and scattering corrections using the ordered subset expectation maximization algorithm (2 interations, 16 subsets) with the point spread function. Image analysis was performed on a dedicated workstation using Extended Brilliance Workspace (ver. 3.5.2.2260, Philips Healthcare). A region of interest (ROI) was set on the whole liver in dynamic axial images while referencing corresponding coronal images and radiating photons were counted over each frame. PET images were subsequently visually evaluated for the presence of focal ^18^F-FDG uptake by radiolabeled *Cs*NEJs. Migration of the *Cs*NEJs to the intrahepatic bile ducts was estimated by semi-quantitatively analyzing photon counts from rabbit liver.

To confirm migration of the *Cs*NEJs to the intrahepatic bile ducts, adult *C. sinensis* were recovered from the liver of the *Cs*NEJ-inoculated rabbits. Four weeks after image scanning, rabbits were euthanized and *C. sinensis* adult worms were recovered from the bile ducts by carefully squeezing liver slices. For pathologic section slides, the liver was fixed in 10% neutral formalin, processed along a routine procedure and stained with hematoxylin and eosin. As a negative control, ^18^F-FDG-labeled *Cs*NEJs were inoculated into two rabbits not injected with CCK-8. In these rabbits, bile is not released from the ampulla of Vater, neither attract the *Cs*NEJs to the bile duct.

## Results

### 1. Labeling efficiency of *Cs*NEJs with ^18^F-FDG

**Figure 2:**
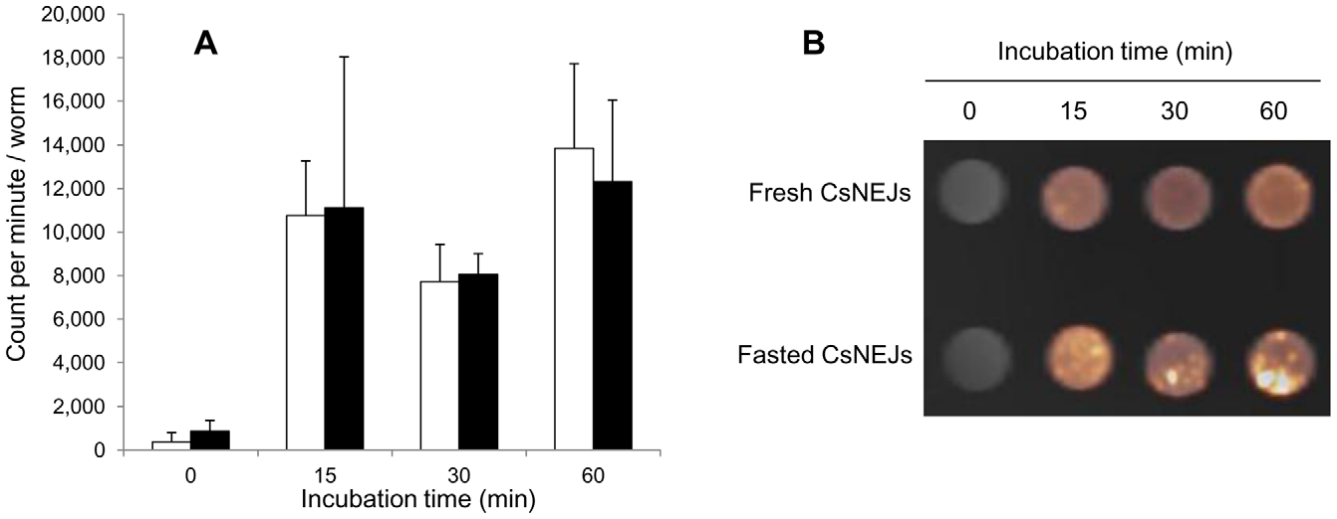
Radiolabeling of newly excysted *C. sinensis* juveniles (*Cs*NEJs) with ^18^F-FDG. A, Radiolabeling efficiency. *Cs*NEJs were radiolabeled freshly after excysted (white) or after fasting for 24 hr (black). B, PET-CT image of *Cs*NEJs labeled with ^18^F-FDG. Labeling efficiencies were not significantly different between the experimental and incubation time groups (*p*>0.05).

Fresh *Cs*NEJs were labeled with 10,760, 7,726 and 13,842 cpm/worm after incubation in radiolabeling media for 15, 30, and 60 min, and fasted *Cs*NEJs were labeled with 11,115, 8,043, and 12,318 cpm/worm when incubated for 15, 30, and 60 min, respectively (Figures 2A & B). Labeling efficiencies were similar in the two groups at all time points. For downstream experiments, fresh *Cs*NEJs were radiolabeled with ^18^F-FDG at 37°C for 15 min.

### 2. Gallbladder contraction and bile release

**Figure 3:**
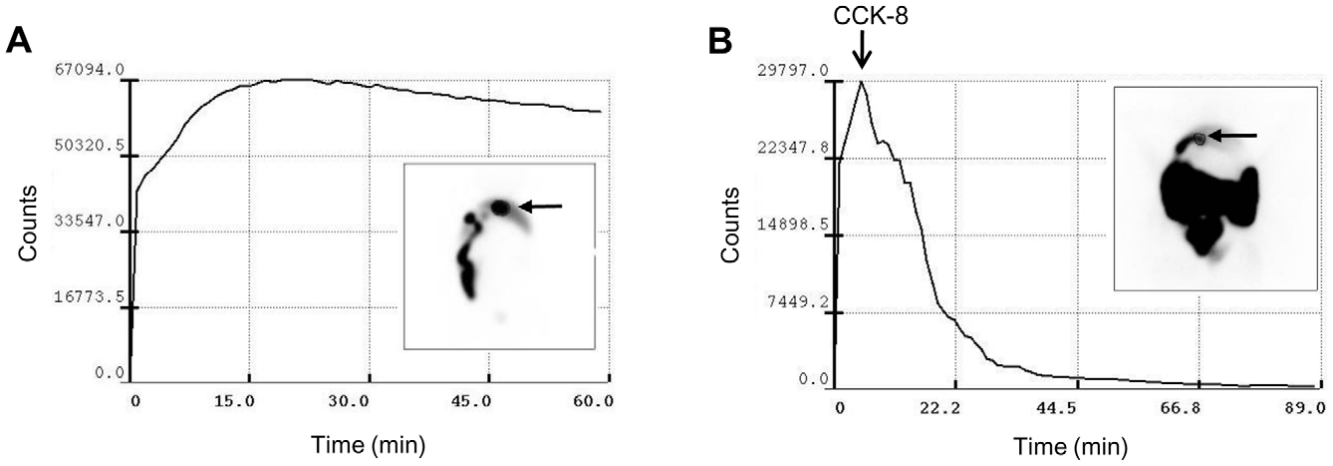
Accumulation and release of bile from rabbit gallbladder by cholescintigraphy and ^99m^Tc-mebrofenin. A, ^99m^Tc-mebrofenin accumulating in gallbladder after intravenous injection. B, CCK-8 triggered gallbladder contraction and bile evacuation. The arrow indicates the gallbladder.

To determine an appropriate time point to inoculate the ^18^F-FDG-labeled *Cs*NEJs in the duodenum after CCK-8 injection, gallbladder contraction and 50% bile emptying times were determined using ^99m^Tc-mebrofenin and cholescintigraphy. After ^99m^Tc-mebrofenin injection, radioactivity increased immediately in the gallbladder to reach a peak at about 15 min, which was maintained for over 60 min (Figure 3A). When rabbits were injected intravenously with CCK-8, ^99m^Tc-mebrofenin was rapidly released from the gallbladder and flowed down the small intestine (Figure 3B). Of the 16 rabbits tested for gallbladder contraction, 6 responded sensitively to CCK-8. On average, it took 11.5 min to evacuate 50% of the gallbladder volume after the first CCK-8 injection. The rabbits responding to CCK-8 were allowed one week to recover and were then included in the *in vivo* imaging experiments.

### 3. Monitoring of *Cs*NEJ migration

Under x-ray visualization and anesthesia, the end of a catheter was located in the mid duodenum (Figure S1). The rabbit was then positioned in the PET-CT bed; anesthesia was maintained with intravenous CCK-8 at a dose of 20 ng/kg every minute during PET-CT scanning. One abdominal CT image was obtained initially and then dynamic PET scanning was started. Three minutes after the initial PET scanning, the ^18^F-FDG-labeled *Cs*NEJs were inoculated into the mid duodenum (Figure 1). Dynamic and static PET scans were carried out using PET-CT on migrating ^18^F-FDG-labeled *Cs*NEJs in 6 rabbits, which included 2 controls.

**Figure 4:**
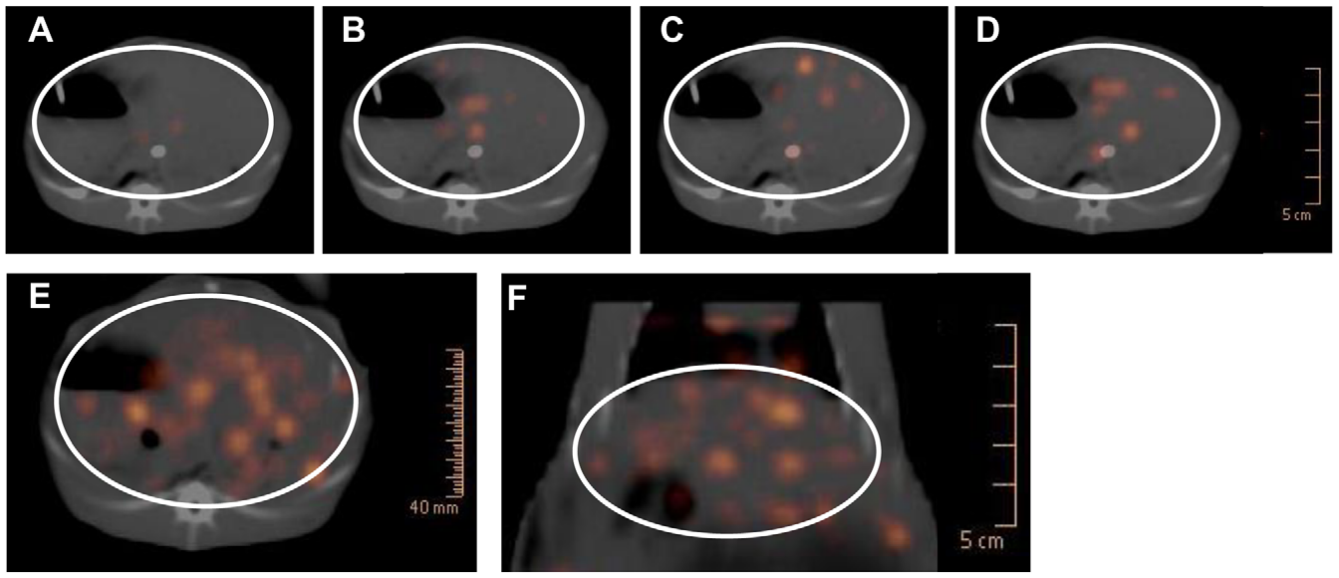
PET-CT images of photons emitted from ^18^F-FDG-labeled *Cs*NEJs in the rabbit liver. Photon images represent *Clonorchis sinensis* arriving at the intrahepatic bile ducts of the rabbit liver. A–D, dynamic images at 7–9, 16–18, 22–24. and 52–54 minutes after *Cs*NEJs inoculation. Static axial (E) and coronal (F) images taken 90 minutes after the *Cs*NEJs inoculation. The liver is located as a region of interest (circle).

Signals emitted from the ^18^F-FDG-labeled *Cs*NEJs were detected in the intestine of the 4 experimental rabbits by PET, and thus, we were able to trace *Cs*NEJ migration by *in vivo* imaging. When the ^18^F-FDG-labeled *Cs*NEJs were injected through the catheter, signals were detected at end of the catheter in the duodenum and along the small intestine driven by peristalsis along the distal portion of the intestine (Figure S2). Signals of *Cs*NEJs appeared in the liver as early as 7–9 min after inoculating the ^18^F-FDG-labeled *Cs*NEJs into the duodenum (Figure 4A).

**Figure 5:**
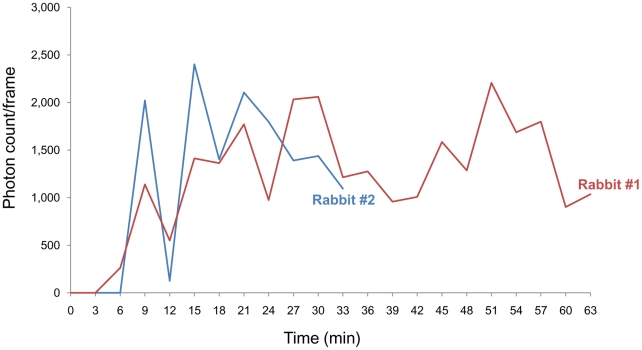
Plot of photon counts from the rabbit liver. The ^18^F-FDG-labeled *Cs*NEJs were attracted with bile juice released by CCK-8-induced gallbladder contraction.

As time elapsed, some photon spots emerged in the liver region and enlarged whereas others faded. These spots appeared to be randomly and evenly distributed in the liver regardless of lobe structure (Figures 4A–D), and gradually increased in number to plateau at about 21 min after inoculation of the radiolabeled *Cs*NEJs (Figures 4 & 5). Spots suggestive of *Cs*NEJs moving through the common bile duct were not observed in PET-CT images. In static PET-CT images taken finally over 10 min, *Cs*NEJs appeared to aggregate in central region of the liver (Figures 4E & F). Of the *Cs*NEJs inoculated into the duodenum, some migrated up to the bile ducts and others down to the lower bowel driven by peristalsis (Figure S2).

In rabbits not injected with CCK-8 (the negative control group), signals of ^18^F-FDG-labeled *Cs*NEJs were only observed in the small intestine in dynamic and static PET images.

At 4 weeks after the *Cs*NEJs inoculation into the duodenum, adult *C. sinensis* worms were found to inhabit and to have provoked pathologic changes in the bile ducts. On average 1,077±806 adults were recovered from the biliary tracts of the rabbits (Figure S3).

## Discussion

*In vivo* the migration route of *C. sinensis* was indirectly determined by ligating the common bile ducts of hosts. Recently, live *Schistosoma mansoni* adults in mice were labeled with protease-activated fluorochrome or ^18^F-FDG and visualized, localized, and quantified using fluorescence molecular tomography or PET [18], [19]. In the present study, we applied the methodologies and investigated PET-CT as a new *in vivo* imaging method for monitoring the migration of *Cs*NEJs and their localization in the rabbit liver. The rabbits are highly susceptible to and retain the *C. sinensis* infections long time to evaluate impact of the infection on the hepatobiliary system. The rabbits have the biliary system similar to that of human. Distribution of *C. sinensis* in the liver of the experimental rabbits was proportional to volume of the liver lobes [20]–[22]. We, therefore, expected the rabbit as a reliable experimental animal model to study bile-chemotactic migration of the *Cs*NEJs, suggesting that findings obtained from the rabbits are applicable to human.

Trematodes import glucose through glucose transporter, and a large number of glucose transporters have registered in the *C. sinensis* transcriptome database [13]. ^18^F-FDG is a glucose analog tagged with isotope ^18^F, and is transported into cytoplasm by glucose transporters in cell membrane. In the cytoplasm, FDG is phosphorylated to FDG-6-phosphate by hexokinase, and FDG-6-phosphate is neither metabolized further nor able to diffuse out of cells. Thus, FDG-6-phosphate is trapped and accumulates in cells as the dephosphorylation of FDG-6-phosphate by glucose-6-phosphatase in cytoplasm is a slow process [15], [23]. We expected that fasted *Cs*NEJs would uptake more FDG than fresh *Cs*NEJs because *Cs*NEJs should have consumed their reserve energy source, primarily glucose, during fasting in glucose-free 1× Locke's solution. However, FDG uptakes in both groups were similar, suggesting that FDG moved quickly into the tegument of *Cs*NEJs through glucose transporter by facilitative diffusion, as was observed for schistosomes [24], [25].

During our studies, we have observed that *Cs*NEJs move toward bile dose-dependently by chemotaxis in *in vitro* assays (unpublished data). Based on our data and the notion that *C. sinensis* juveniles migrate up through the common bile duct, it was essential that bile juice is released from the gall bladder to attract *Cs*NEJs into the common bile duct.

Technetium labeled hepatobiliary radiopharmaceuticals has greatly facilitated studies on gallbladder function [26]. Since CCK-stimulated cholescintigraphy was first reported in 1979, gallbladder emptying function has been measured by using standard cholagogic stimulus agents by biliary excretion scintiography [27], [28]. Cholescintigraphy with ^99m^Tc-iminodiacetic acid has been used to diagnose diseases in the biliary system, such as, bile duct obstruction, cholelithiasis, cholecystitis, and biliary fistula [29]–[31].

The gallbladder normally fills with hepatic bile during fasting and empties its contents into the duodenum in response to stimulation by CCK, either released endogenously following a meal or administered exogenously [32]. However, gallbladder emptying response to exogenous CCK varies among patients and experimental animals. In this study, gallbladder contraction and bile juice release was achieved by repeatedly injecting CCK-8. By cholescintigraphy, ^99m^Tc-mebrofenin was found to be released rapidly from gallbladders after CCK-8 administration. Thus, this scheme enabled us to study *in vivo* bile-chemotactic behavior of *Cs*NEJs in rabbits.

Using *Cs*NEJ radiolabeling and bile excretion from gallbladder, images of *Cs*NEJs migrating to the intrahepatic bile ducts in rabbits were obtained by PET-CT. The radiolabeled *Cs*NEJs were inoculated into the mid duodenum, which is supposed to be an excystation site for *C. sinensis* metacercariae [3], [6]. We visualized ^18^F-FDG-labeled *Cs*NEJs migrating to the liver in experimental rabbits using PET-CT. The first signals of *Cs*NEJs arriving at the liver from the duodenum were detected by dynamic PET as early as 7–9 min after inoculating *Cs*NEJs into duodena. At 21 minutes post-inoculation, photon signals emitted from *Cs*NEJs in liver appeared to have stabilized though their intensities undulated, which suggested most *Cs*NEJs responsive to bile immediately migrated up to the intrahepatic bile duct. Imaging was ended with a final static PET-CT image because signals were of greater intensity than on dynamic PET images, suggesting that some *Cs*NEJs were late to arrive and accumulated in the intrahepatic bile ducts [3]. In *in vitro* assays, *Cs*NEJs showing rapid bile-taxis were promptly re-activated and moved rapidly and continuously toward bile added to assay chambers, and slow responders responded slowly (unpublished data).

The artificial manipulation of *Cs*NEJs employed in this study, that is, *in vitro* excystation and radiolabeling, and inoculation into the duodenum, may have reduced adaptation to body temperature, chemotactic response to bile, migration to the bile duct, and survival in bile juice. To compensate for this, in the present study, 3–5 times more ^18^F-FDG-*Cs*NEJs than normally usual experiment was inoculated via catheter into the duodenum. We believe that slow responders arrived late at the intrahepatic bile ducts after PET scans, and increased numbers of *C. sinensis* adult worms recovered from the bile ducts [3]. When filet of the fresh water fish was minced by teeth and ingested by mammalian animals including human, the *C. sinensis* metacercariae could be released from the filet in the stomach after 1–2 hour, and then passed down to the duodenum. Considering immediate excystation of the *C. sinensis* metacercariae in contact with trypsin [6], human infection may take place within 2–3 hours after eating raw filet of the fresh water fish.

We searched for photonic signals from the common bile ducts in dynamic and static PET-CT images of experimental rabbits, but found no signal. The common bile duct is narrow and *Cs*NEJs either passed rapidly or steadily in file, and thus, only small number of juveniles (not enough to create a PET-CT image) was captured in a given frame. Furthermore, anatomically the common bile duct is located in the deep abdomen under the liver, which hinders emitted photons.

Collectively, we report for the first time that *Cs*NEJs were efficiently radiolabeled *in vitro* with ^18^F-FDG, and that *Cs*NEJs migrate quickly with bile-chemotaxis to the intrahepatic bile duct as visualized in rabbits by PET-CT.

## Supporting Information

Figure S1 **Insertion of a catheter into the mid duodenum of a rabbit under anesthesia.**
(TIF)

Figure S2 **PET-CT coronal images showing some ^18^F-FDG-labeled** ***Cs*** **NEJs driven down the small intestine by peristalsis.** A–D, 9, 18, 24, and 27 minutes after inoculating radiolabeled *Cs*NEJs into the mid duodenum.
(TIF)

Figure S3 ***Clonorchis sinensis*** **from an experimental rabbit liver 4 weeks after a bile-chemotaxis experiment.** A, Adult flukes in the rabbit liver, hematoxylin-eosin stained. B, Adult flukes recovered from the rabbit's liver.
(TIF)
