# Supplementary figures and images for: Tracing of the Bile-Chemotactic Migration of Juvenile Clonorchis sinensis in Rabbits by PET-CT
Source: PLoS Negl Trop Dis. 2011 Dec 13;5(12):e1414. doi: 10.1371/journal.pntd.0001414 (PMC3236719; doi:10.1371/journal.pntd.0001414)

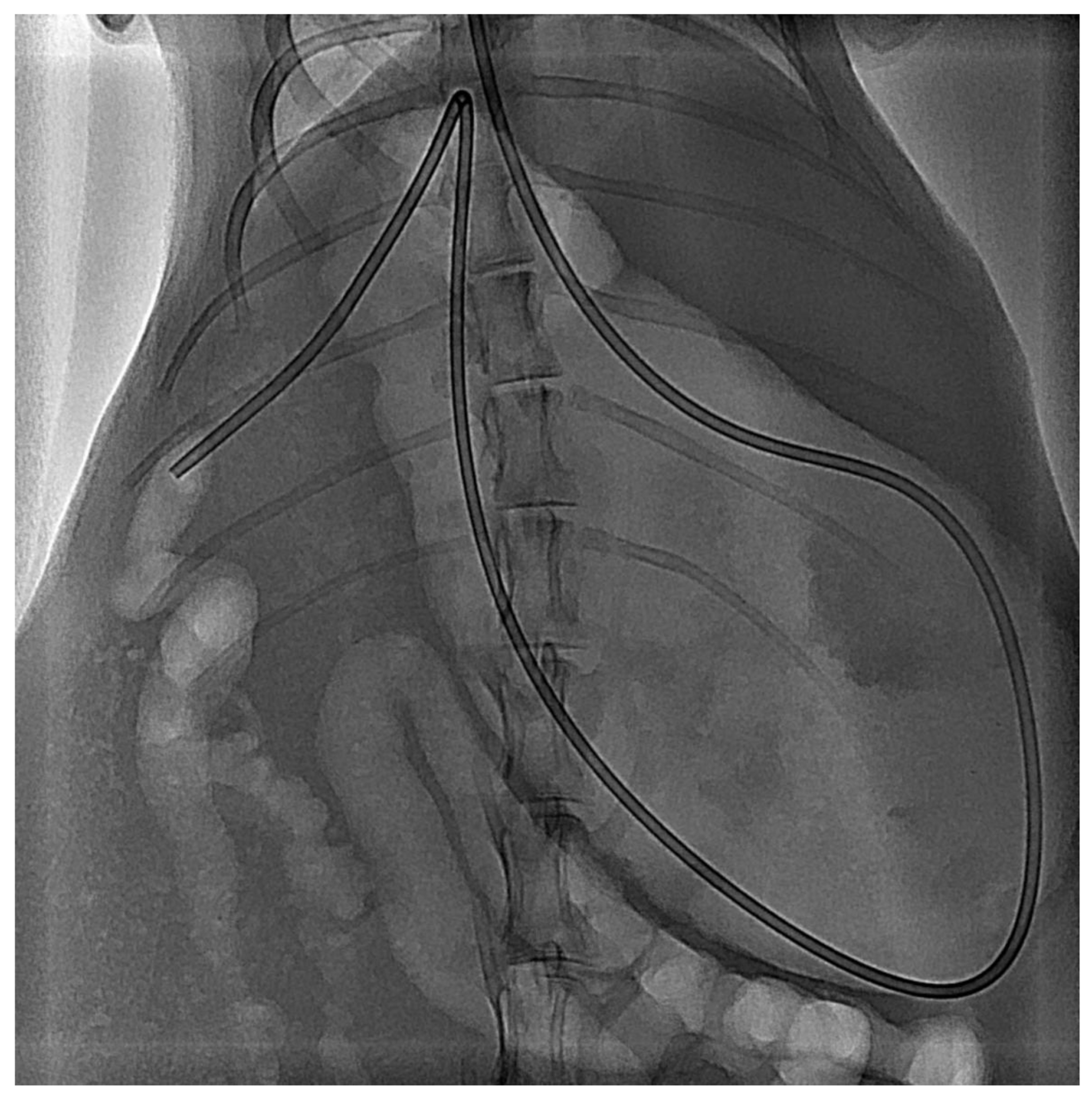

Supplement: Figure S1 — Insertion of a catheter into the mid duodenum of a rabbit under anesthesia. (TIF) [file pntd.0001414.s001.tif]

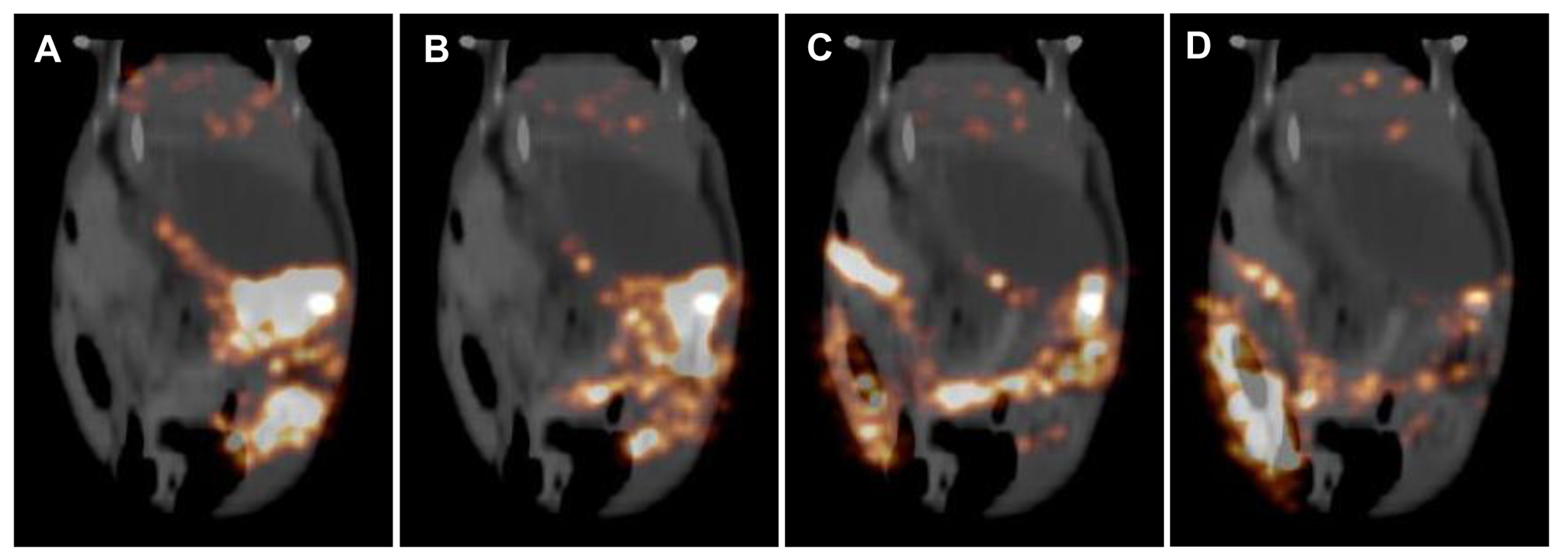

Supplement: Figure S2 — PET-CT coronal images showing some 18F-FDG-labeled Cs NEJs driven down the small intestine by peristalsis. A–D, 9, 18, 24, and 27 minutes after inoculating radiolabeled CsNEJs into the mid duodenum. (TIF) [file pntd.0001414.s002.tif]

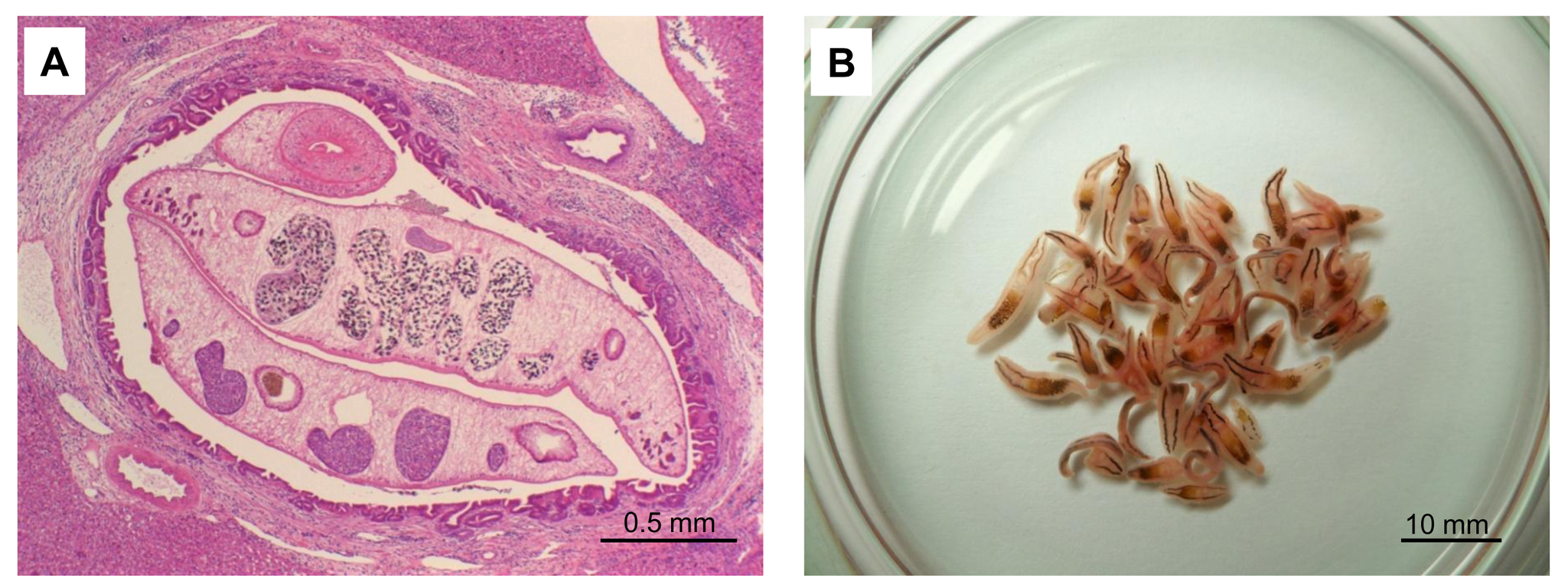

Supplement: Figure S3 — Clonorchis sinensis from an experimental rabbit liver 4 weeks after a bile-chemotaxis experiment. A, Adult flukes in the rabbit liver, hematoxylin-eosin stained. B, Adult flukes recovered from the rabbit's liver. (TIF) [file pntd.0001414.s003.tif]
